# An All-in-One Testing Chip for the Simultaneous Measurement of Multiple Thermoelectric Parameters in Doped Polysilicon

**DOI:** 10.3390/mi16020116

**Published:** 2025-01-21

**Authors:** Lei Shi, Na Zhou, Jintao Wu, Meng Shi, Yizhi Shi, Cheng Lei, Haiyang Mao

**Affiliations:** 1 Key Laboratory of Micro/Nano Devices and Systems, North University of China, Ministry of Education, Taiyuan 030051, China; 2 Institute of Microelectronics of the Chinese Academy of Sciences, Beijing 100029, China; 3 University of Chinese Academy of Sciences, Beijing 100049, China

**Keywords:** thermoelectric parameters, all-in-one testing chip, polysilicon, Seebeck coefficient, thermal conductivity

## Abstract

Polysilicon is widely used as a thermoelectric material due to its CMOS compatibility and tunability through doping. The accurate measurement of the thermoelectric parameters—such as the Seebeck coefficient, thermal conductivity, and electrical resistivity—of polysilicon with various doping conditions is essential for designing and fabricating high-performance thermopile sensors. This work presents an all-in-one testing chip that incorporates double-layer thermoelectric structures on a suspended membrane-based supporting layer, with polysilicon constituting at least one of these thermoelectric layers. By employing a differential calculation approach in conjunction with thermal imaging methods, we could simultaneously measure various thermoelectric parameters—including resistivity, the Seebeck coefficient, and thermal conductivity—of polysilicon under different doping conditions. Furthermore, the method proposed in this study provides a means for accurately obtaining thermoelectric parameters for other materials, thereby facilitating the design and optimization of thermoelectric devices.

## 1. Introduction

Thermopile infrared sensors, which are based on the thermoelectric effect, offer several advantages such as non-contact detection, uncooling, strong environmental adaptability, and low cost. As a result, they have been widely used in various fields including military reconnaissance, industrial control, aerospace, and medical detection [1,2,3]. Recently, with the development of micromachining technologies, due to their CMOS process compatibility [4,5], thermopile infrared sensors have become a research hotspot [6,7,8]. In recent years, advancements in micromachining technologies have further enhanced the appeal of thermopile infrared sensors, particularly due to their compatibility with CMOS processing [4,5]. Among the thermoelectric materials used in these sensors, polysilicon is the most frequently employed due to its ability to be implanted with n- and p-type dopants at various concentrations, allowing for a significant and tunable difference in the Seebeck coefficient. Additionally, the preparation process for polysilicon is compatible with CMOS standards, making it suitable for large-scale production [9,10].

The thermoelectric conversion efficiency of a material is typically assessed by its thermoelectric merit [11,12,13], which is critical for achieving high-performance thermopiles. To maximize the thermoelectric merit, it is essential to attain a large Seebeck coefficient, low thermal conductivity, and low electrical resistivity; these three parameters are fundamental for ideal thermoelectric materials used in thermopile infrared sensors [14]. In the design and optimization of thermopile infrared sensors, it is of great significance to measure the thermoelectric parameters, including resistivity, the Seebeck coefficient, and thermal conductivity, of polysilicon films with varying doping concentrations, structures, and dimensions.

For resistivity measurement, the Van de Pauw structure is commonly used [15]. On the other hand, to measure the Seebeck coefficient and the thermal conductivity, several testing structures have been proposed. For instance, Xie, J. et al. reported a planar test structure (located on the surface of a silicon substrate, requiring no release of the substrate) [15], in which the hot end of a doped polysilicon sample is equipped with a microheater for temperature control, while two thermistors are positioned at both the hot and cold ends for temperature measurement. Such a testing structure was further utilized by Li, Y. et al. and Kang, T. to measure the Seebeck coefficient of doped polysilicon [16,17]. Although the planar testing structure has relatively simple construction and is relatively easy to prepare, in such a structure, a significant amount of heat dissipates into the substrate, which leads to substantial measurement errors, making it unsuitable for measuring the parameter of thermal conductivity.

To avoid heat dissipation through the substrate, and thus to address the challenges, Zhou, H. et al. and Huang, P. et al. [18,19,20] also reported cantilever-based testing structures capable of measuring both the Seebeck coefficient and thermal conductivity. Compared to planar structures, cantilever-based designs allow heat to propagate directionally along its structure, resulting in a larger temperature difference between its two ends, which enhances measurement accuracy. However, real-time temperature measurement necessitates that a thermistor is suspended at the suspended end of the cantilever, which also requires calibration prior to use and thus significantly increases the workload. Furthermore, the preparation of cantilever-based structures requires back-cavity etching and suspension film-front etching, which are more difficult.

This work presents an all-in-one testing chip featuring double thermoelectric layers positioned on a suspended supporting membrane, fabricated through a simple process. Within the chip, polysilicon serves as the material for at least one of these thermoelectric layers, enabling differential calculations to simultaneously obtain three thermoelectric parameters from various materials. This structure eliminates the impact of the supporting layer’s thermal conductivity by stacking different thermocouple materials. Utilizing differential calculations and a thermal imaging approach, the resistivity, Seebeck coefficient, and thermal conductivity of doped polysilicon can be measured simultaneously and with high accuracy. The chip structure is compact, and the measurement and calculation methods significantly streamline the testing process. Furthermore, the novel testing chip is fabricated using the same procedures as those for double-layer MEMS thermopiles, which offer enhanced stability compared to cantilever-based structures. The innovative method introduced in this work provides important guidance for the design and optimization of thermoelectric devices.

## 2. Materials and Methods

### 2.1. Structural Design and Fabrication

**Figure 1:**
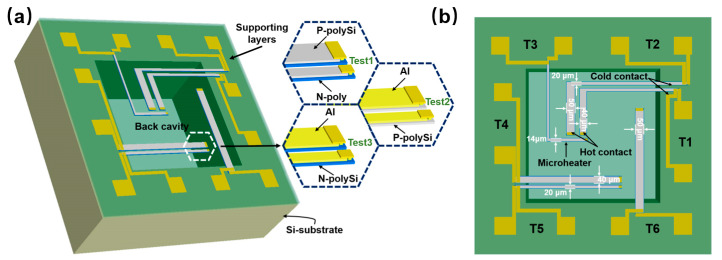
A schematic diagram of the suspended, membrane-supported, double-layer differential testing structures distributed in one chip: (**a**) the 3D structure; (**b**) a top view and the dimensions of the structures.

Figure 1 illustrates the suspended, membrane-supported, double-layer differential testing structures, composed of a silicon substrate, a supporting layer, double-layer thermocouples, and aluminum electrodes. To assess the thermoelectric parameters of N-doped polysilicon (N-polySi) and P-doped polysilicon (P-polySi), stacked structures with different thermocouple materials—specifically N-polySi, P-polySi, and aluminum—were designed and labeled as Test1, Test2, and Test3, respectively. In Test1, the double-layer thermocouples were composed of N-polySi and P-polySi. Test2 features P-polySi and aluminum, while Test3 incorporated N-polySi and aluminum (Figure 1a).

For all three types of structures, the silicon substrates were etched from the backside to create back cavities, thereby suspending the supporting layers. Each chip contained six pairs of thermocouples on the supporting layers, designated as T1, T2, T3, T4, T5, and T6, along with 12 pads (Figure 1b). T1 and T2 were utilized to measure the Seebeck coefficient and thermal conductivity of N-polySi and P-polySi. They were arranged in an “L” shape, which increased the distance between the hot and cold ends, thereby enhancing the temperature difference between the two ends. Notably, T3 was situated near the hot ends of T1 and T2, serving as a microheater in the experiment. When supplied with voltage, it generated a temperature difference across the T1 and T2 thermocouples.

Additionally, T4, T5, and T6 had the same length but different widths of 40 µm, 20 µm, and 50 µm, respectively, and were employed to measure the resistivity of N-polySi and P-polySi. The stacked materials and the corresponding functions of T1 to T6 are summarized in Table 1.

The feasibility of the designed structure was analyzed using the finite element simulation software COMSOL Multiphysics (6.1). First, a suspended membrane-based structure and a cantilever-based structure were created using SolidWorks (2021). The Structural Mechanics Module in COMSOL Multiphysics (6.1) was then employed to analyze the stress distributions on the supporting layers of both structures. In the simulation, a fixed constraint was applied to the bottom of the chip, and a gravitational load was also applied to the entire chip. Subsequently, strain distributions on the testing structures were calculated. Next, the Heat Transfer Module and the AC/DC Module were utilized to simulate temperature and potential distributions. In these simulations, the temperature at the bottom of the chip was set to 273.15 K, and a voltage of 0.5 V was applied to T3.

**Figure 2:**
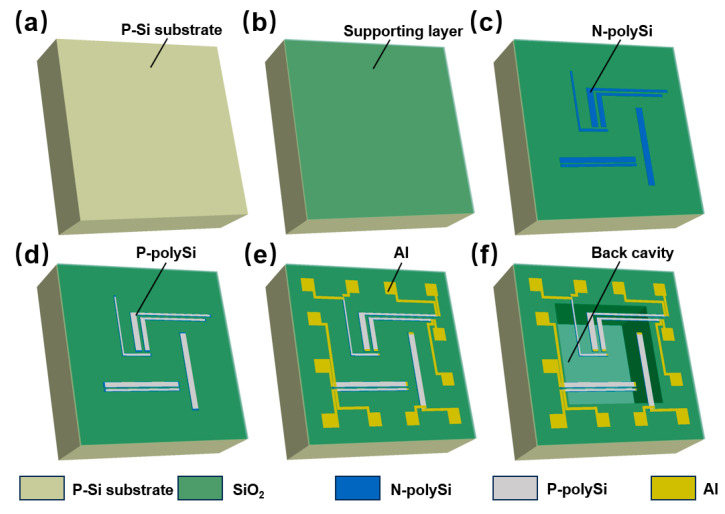
The fabrication process of the suspended, membrane-supported, double-layer differential testing structures (Test1 is taken as the example): (**a**) A silicon wafer served as the substrate; (**b**) Depositing SiO_2_-Si_3_N_4_-SiO_2_ composite film; (**c**) Forming N-polySi strips. (**d**) Forming P-polySi strips. (**e**) Sputtering Al to form electrical connection, electrodes. (**f**) Structural release at backside.

Figure 2 illustrates the fabrication process of the suspended, membrane-supported, double-layer differential testing structures, which was compatible with CMOS technology. First, a silicon wafer served as the substrate (Figure 2a), onto which a SiO_2_-Si_3_N_4_-SiO_2_ composite film (with an overall thickness of 1.3 μm) was deposited as the supporting layer using low-pressure chemical vapor deposition (LPCVD) (Figure 2b). Next, a 420 nm-thick polySi film was deposited on the supporting layer, followed by the injection of phosphorus ions (at a dose of 1 × 10^16^ cm^−2^) into the polySi film. N-polySi thermocouple strips were then formed using photopatterning and dry etching (Figure 2c). Subsequently, a SiO_2_ insulating layer was deposited on the N-polySi thermocouple strips, followed by a second layer of polySi (300 nm thick), and injected with boron ions (also at a dose of 1 × 10^16^ cm^−2^) and annealed (1050 °C, 30 s). Following photolithography, P-polySi thermocouple strips were prepared (Figure 2d). This process resulted in the formation of double-layer thermocouples composed of N-polySi and P-polySi strips, which were designated as Test1 after the two strips were connected by aluminum. Another SiO_2_ layer was then deposited, and holes were opened in this layer. An aluminum layer (e.g., 300 nm) was subsequently sputtered and patterned on the wafer to form the connecting lines and the second layer of thermocouple strips for Test2 and Test3 (Figure 2e). Finally, a back cavity was created by dry etching from the rear, utilizing the SiO_2_-Si_3_N_4_-SiO_2_ composite film as a self-stop layer (Figure 2f).

### 2.2. Experimental Setup

**Figure 3:**
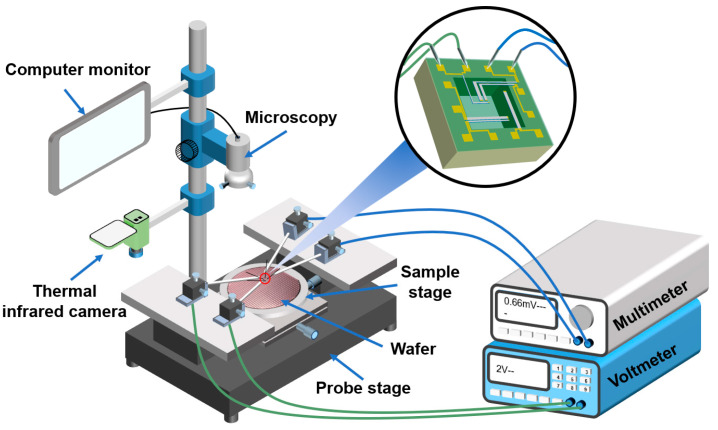
A diagram of the system for testing thermoelectric parameters using the prepared testing structures.

Figure 3 illustrates the schematic diagram of the system used for testing thermoelectric parameters with the prepared double-layer testing structures. The system consisted of a probe stage (MMS-06, Alite Semitech, Hong Kong, China) for applying and measuring electrical parameters, a thermal infrared camera (DS-2TP96-25SQF/W, Hikvision, Hangzhou, China) to measure the temperatures at both ends of T2, a voltmeter (UNI-T-UDP3303C, UNI-T, Guangdong, China) for voltage supply, and a multimeter (KEITHLEY-DMM6500, KEITHLEY, Beijing, China) for measuring electrical parameters. The resistivities of the materials were determined based on the I-V characteristic curves of T4, T5, and T6. To test the Seebeck coefficients and thermal conductivities of the three materials, a voltage was applied to the microheater using the voltmeter, providing heat to the hot ends of T1 and T2. The voltage signals converted from each thermocouple were recorded by the multimeter, while the thermal infrared camera measured the temperatures at both the hot and cold ends of the thermocouples.

## 3. Results

### 3.1. Fabrication of Testing Structures

**Figure 4:**
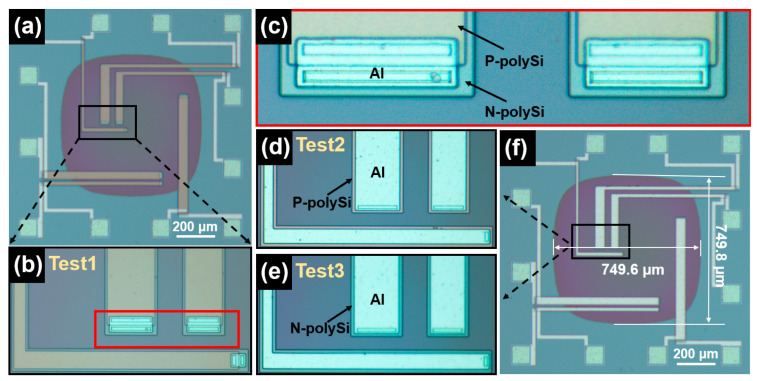
Optical images of the suspended, membrane-supported, double-layer differential testing structures after preparation: (**a**) Successfully prepared testing structures; (**b**) Test1; (**c**) Enlarged view of Test1; (**d**) Test2; (**e**) Test3; (**f**) Back cavity dimensions of the testing structures.

Figure 4 presents optical images of the prepared testing structures. Through three steps of photolithography, three testing structures, each stacked with different thermoelectric materials, were successfully fabricated on separate chips from the same wafer, with a back cavity incorporated into each chip (Figure 4a). These structures are referred to as Test1 (Figure 4b), Test2 (Figure 4d), and Test3 (Figure 4e), with cavity dimensions of 749.8 μm × 749.6 μm (Figure 4f). In the amplified image of Test1, the N-polySi on the lower layer appears light-blue, while the P-polySi on the upper layer is light-yellow (Figure 4c).

### 3.2. Simulation Results

**Figure 5:**
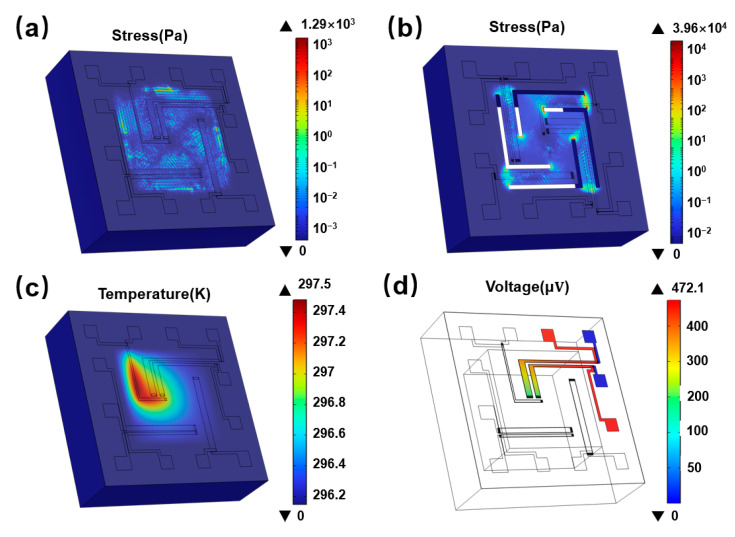
(**a**,**b**) Simulated stress distributions in a suspended, membrane-based structure and a cantilever-based structure. (**c**) Temperature distribution. (**d**) Potential distributions in suspended membrane-based structures when a 0.5 V voltage was applied to T3.

Figure 5 presents the simulation results. The maximum film stress on the suspended membrane-based structure was 1.29 kPa (Figure 5a), while the stress on the cantilever-based structure was significantly higher at 39.60 kPa (Figure 5b). Applying a voltage of 0.5 V to T3 resulted in a temperature increase of 1.3 K at the hot end of T2 (Figure 5c), and the temperature distribution was triangular due to the accumulation of heat at the corners of the heating resistor. Consequently, the thermoelectric effect generated a voltage of 472.1 µV in T2 (Figure 5d).

### 3.3. Characterization

#### 3.3.1. Resistivity of Doped PolySi

Utilizing thermoelectric materials with low resistivity can decrease the internal resistance of thermoelectric devices, thereby reducing noise [21]. This reduction in internal resistance simultaneously enhances detectivity, as detectivity is inversely proportional to noise. In thermopile array sensors, excessive internal resistance in the pixels can lead to thermal crosstalk, significantly affecting imaging results [22,23,24]. Therefore, measuring the resistivity of polySi is crucial when it is employed as a thermoelectric material.

**Figure 6:**
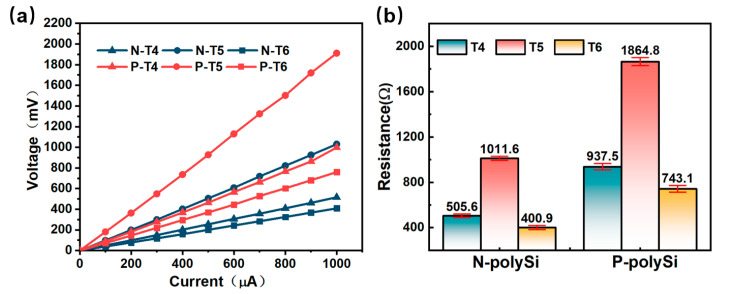
(**a**) I-V characteristic curves of T4, T5, T6. (**b**) Resistances of T4, T5, and T6 of different types.

Figure 6a presents the resistance and I-V characteristic curves for T4, T5, and T6. As the input current increased, the voltage across the thermocouples increased linearly. The resistances of T4, T5, and T6 were derived from the slopes of these curves. Given the relationship among the widths, defined as WT5 = 1/2 WT4 = 2/5 WT6, it follows that RT5 = 2RT4 = 5/2RT6. As illustrated in the inset of Figure 6b, the resistances of the N-polySi for T4, T5, and T6 were measured to be 505.6, 1011.6, and 400.9 Ω, respectively, which agrees well with the derived relationship, RT5 = 2RT4 = 5/2RT6. Similarly, the resistances of T4, T5, and T6 using P-polySi were obtained as 937.5, 1864.8, and 743.1 Ω, respectively.

Using the resistance formula $R=\gamma \frac{l}{d\cdot w}$ (γ is the resistivity, and l, w, and d are the length, width, and height of the measured structures), the resistivities of N-polySi and P-polySi with specific doping conditions in this work were calculated to be 1.415 × 10^−5^ Ω·m and 2.625 × 10^−5^ Ω·m, respectively. The difference in resistivities between the two types could be attributed to the varying mobilities of the charge carriers.

#### 3.3.2. Seebeck Coefficient of Doped PolySi

**Figure 7:**
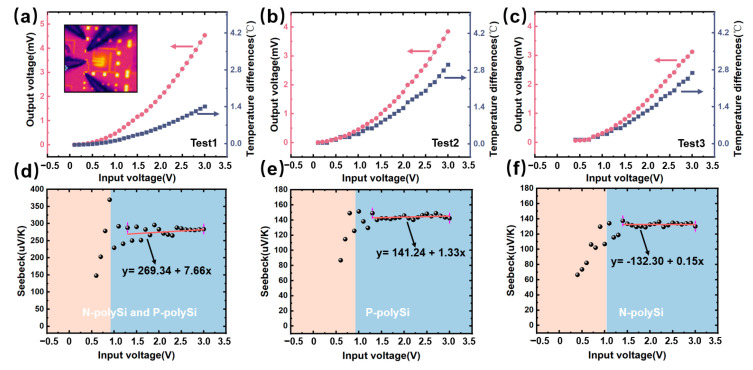
(**a**–**c**) The output voltage curves and temperature difference curves of T2 in Test1, Test2, and Test3 with respect to different input voltages at T3. (**d**–**f**) The Seebeck coefficients of P-polySi and N-polySi, P-polySi, N-polySi.

Figure 7a–c demonstrate the output voltage curves and temperature difference curves of T2 in Test1, Test2, and Test3 with respect to different input voltages at T3. To calculate the Seebeck coefficient difference α based on one thermocouple strip, it can be expressed as

(1) $$\alpha =\frac{U}{T_{h}-T_{c}}$$

Here, U refers to the output voltage of T2, which can be obtained from the red lines in Figure 7a–c. Th and Tc refer to the temperatures at hot end and cold end of T2, which can be obtained from the thermal images, as shown in inset of Figure 7a; accordingly, the temperature difference in (Th − Tc) can be obtained from the gray lines in Figure 7a–c as well. Since there were different material stacks in the testing structures of Test1, Test2, and Test3, the Seebeck coefficient differences between two different stacking materials could be obtained, and as the Seebeck coefficient of Al was already known as −3.2μV/K, the Seebeck coefficient difference between N-polySi and P-polySi could be calculated from Test1, and it was 259.34 μV/K. The Seebeck coefficient of P-polySi was calculated from Test2, and it was 141.24 μV/K, and N-polySi was calculated from Test3, which was −132.30 μV/K.

#### 3.3.3. Thermal Conductivity of Doped PolySi

The thermal conductivity of a thermoelectric material is inversely proportional to the temperature difference between the two ends of the thermoelectric structure; therefore, it also has influence on the performance of the thermopile sensors. Thus, is worth investigating and testing.

When a voltage, V, is applied to a microheater (e.g., T3 in the chip), the heating power P of the microheater is $P=\frac{V^{2}}{R_{T3}}$. In such a case, the temperature difference at both ends of T2 is $∆T=T_{h}-T_{c}$, which can be obtained from Figure 7a–c. Herein, there is a relationship between the heating power P and ∆T, which is expressed as

(2) P=G·∆T

where G represents the overall solid thermal conductance of the structure, including the thermal conductance of the stacked thermocouples, of the supporting layer ($G_{Supporting\;layer}$) and of the dielectric layer ($G_{Dielectric\;layer}$) as well. Therefore, for the testing structures in Test1, the thermal conductance can be listed as

(3) $$G_{Test1}=G_{N-polySi}+G_{P-polySi}+G_{Supporting\;layer}+G_{Dielectric\;layer}$$

Specifically, the solid thermal conductance for each part can be expressed as

(4) $$G_{i}=\frac{\lambda_{i}d_{i}w_{i}}{l_{i}}$$

Among them, $\lambda_{i}$, $d_{i}$, $w_{i}$, and $l_{i}$ are the thermal conductivity, the thickness, the length, and the width of the structure being studied. Similarly, for those in Test2, there is

(5) $$G_{Test2}=G_{P-polySi}+G_{Al}+G_{Supporting\;layer}+G_{Dielectric\;layer}$$

For those in Test3, there is

(6) $$\;G_{Test3}=G_{N-polySi}+G_{Al}+G_{Supporting\;layer}+G_{Dielectric\;layer}$$

Since the testing structure Tn in Test1, Test2, and Test3 had the same dimensions and the same supporting layers and dielectric layers, the only difference among them was the different thermocouple materials; therefore, we assumed that $G_{Supporting\;layer}$ and $G_{Dielectric\;layer}$ were equal in Test1, Test2, and Test3. In addition, the thermal conductivity of Al was already known as 237 W/(m·K). By taking the dimensions of T2 into consideration, $G_{Al}$ = 52.7 × 10^−5^ W/K was calculated according to Equation (2). Then, with a voltage applied to T3, the heating power P could be obtained according to the aforementioned method. In addition, the temperature difference between the hot and cold ends of T2 was measured using a thermal infrared camera. According to Equation (4), $G_{Test1}$, $G_{Test2}$, and $G_{Test3}$ could be calculated separately. Then, with Equations (3), (5) and (6), $G_{N-polySi}$ and $G_{P-polySi}$ could be obtained by differential calculation.

**Figure 8:**
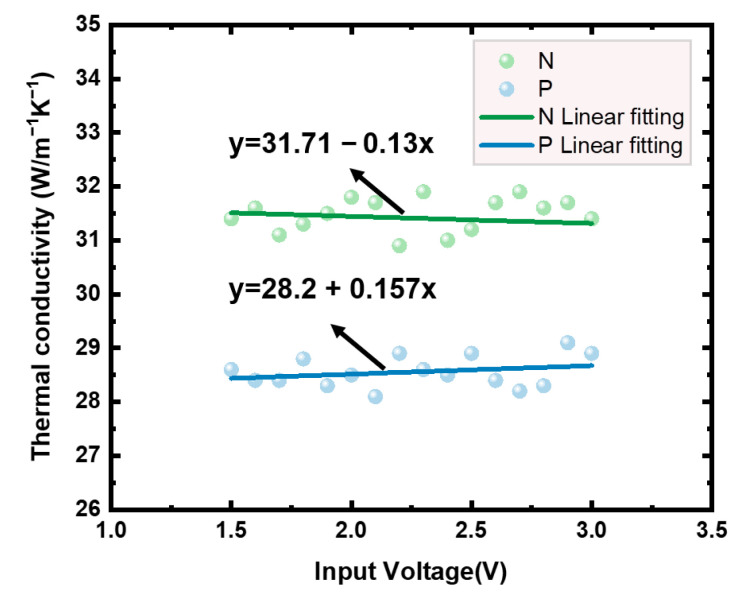
Thermal conductivity curves of N-polySi and P-polySi.

Finally, according to Equation, the thermal conductivities of N-polySi and P-polySi with the specific doping conditions were $\lambda_{N-PolySi}=31.7\;W/(m\cdot K)$ and $\lambda_{P-PolySi}=28.2\;W/(m\cdot K)$, as shown in Figure 8.

## 4. Conclusions

In this study, double-layer differential testing structures incorporating various stacks of thermocouple materials were designed and fabricated on a chip. Using a differential calculation approach, the resistivities, Seebeck coefficients, and thermal conductivities of P-polySi and N-polySi thermocouple strips were determined. For the doping conditions utilized in this work, the resistivities of N-polySi and P-polySi were 1.415 × 10^−5^ Ω·m and 2.625 × 10^−5^ Ω·m, respectively. The Seebeck coefficients were −132.3 μV/K for N-polySi and 141.24 μV/K for P-polySi, while the thermal conductivities were 31.71 Wm^−1^K^−1^ for N-polySi and 28.20 Wm^−1^K^−1^ for P-polySi. This testing method can also be applied to other thermoelectric materials and can be conducted using the same procedures as those for thermoelectric devices, allowing for the accurate measurement of the thermoelectric properties of materials to inform the design and optimization of such devices.

## Figures and Tables

**Table 1:** Parameters and functions of different testing structures.

	Dimensions (mm) (l × w × d)	Materials in Test1	Materials in Test2	Materials in Test3	Functions
T1	(263 × 40 + 514 × 20) × 0.42 (260 × 36 + 503 × 16) × 0.3	N-polySi and P-polySi	P-polySi and Al	N-polySi and Al	For measuring α and λ
T2	(299 × 50 + 680 × 20) × 0.42 (296 × 46 + 665 × 16) × 0.3	N-polySi and P-polySi	P-polySi and Al	N-polySi and Al	For measuring α and λ
T3	670 × 15 × 0.42 662 × 11 × 0.3	N-polySi and P-polySi	P-polySi and Al	N-polySi and Al	As a microheater
T4	592 × 20 × 0.42 579 × 16 × 0.3	N-polySi and P-polySi	P-polySi and Al	N-polySi and Al	For measuring γ
T5	592 × 40 × 0.42 579 × 36 × 0.3	N-polySi and P-polySi	P-polySi and Al	N-polySi and Al	For measuring γ
T6	592 × 50 × 0.42 579 × 48 × 0.3	N-polySi and P-polySi	P-polySi and Al	N-polySi and Al	For measuring γ

α refers to the Seebeck coefficient; λ refers to the thermal conductivity; and γ is the resistivity.

## Data Availability

The raw data supporting the conclusions of this article will be made available by the authors on request.
